# The deubiquitination enzyme USP14 promotes the tumourigenesis of gastric cancer by enhancing c-MYC nuclear translocation through deubiquitination of KPNA2

**DOI:** 10.1038/s41419-025-08065-2

**Published:** 2025-10-21

**Authors:** Jia Li, Houyi Tang, Hongbo Chang, Yi Du, Liang Du, Erhu Zhao

**Affiliations:** 1 Beijing Forestry University, 100083 Beijing, China; 2 State Key Laboratory of Resource Insects, Medical Research Institute, Southwest University, Chongqing, 400716 China; 3 Jinfeng Laboratory, Chongqing, 401329 China

**Keywords:** Cancer, Cancer therapy

## Abstract

The deubiquitinating enzyme USP14, which belongs to the ubiquitin-specific protease family, is highly expressed in various malignant tumors. The regulatory mechanisms in tumors are complex and diverse, encompassing a range of cellular processes such as proliferation, apoptosis, inflammation, autophagy, and drug resistance. However, the functional role of USP14 in gastric cancer remains unclear. In the current investigation, a significant upregulation of USP14 expression was observed in gastric cancer, and its overexpression was associated with an unfavorable prognosis among patients. The involvement of USP14 is indispensable for promoting the growth, motility, and infiltration of gastric cancer cells, as revealed by our findings. Further investigations revealed that USP14 interacts with KPNA2 and is responsible for deubiquitinating it by removing ubiquitin. Moreover, the deubiquitylation process mediated by USP14 was found to be critically dependent on the K48 residue of ubiquitin. The knockdown of USP14 significantly suppressed the proliferation, migration, and invasion of gastric cancer cells. This effect was attributed to the regulation of c-MYC nuclear translocation through KPNA2 deubiquitination. The findings underscore the imperative for further evaluation of the potential therapeutic significance of USP14 in gastric cancer.

## Introduction

Globally, gastric cancer (GC) ranks among the top five most prevalent malignancies and represents the third leading cause of cancer-related mortality. Annually, there are nearly 1 million incident cases of stomach cancer worldwide, resulting in approximately 700,000 deaths [1, 2]. The rate of metastasis in gastric cancer is among the highest compared to other types of malignant tumors, resulting in a significantly low five-year survival rate for patients with metastatic gastric cancer. The lymph, liver, and lung are the main target organs of gastric cancer metastasis, and it can also transfer to other parts through blood metastasis at a late stage. Inhibiting or reducing metastasis of gastric cancer has become a key link in the treatment of GC [3, 4]. The exploration of the specific molecular mechanism behind gastric cancer metastasis can help identify more accurate bio-markers, which is crucial for designing improved treatment plans and enhancing patient prognosis and survival rates [5].

Ubiquitin-specific protease 14 (USP14) is a member of the ubiquitin-specific proteases (USPs) family, consisting of an N-terminal ubiquitin-like (UBL) domain and a C-terminal catalytic USP domain. USP14 is expressed in multiple normal tissues, where it regulates proteasomal degradation and cellular homeostasis. The level of the substrate protein can be stabilized by removing the ubiquitin label of the substrate protein, which is involved in a variety of signaling pathways and determines the fate of the cell [6]. USP14 has been shown to preferentially cleave K48-linked polyubiquitin chains, which typically target proteins for proteasomal degradation, while showing less activity toward K63-linked chains [7, 8]. Known USP14 substrates include β-catenin, vimentin, androgen receptor, and cell cycle regulators, highlighting its diverse cellular roles. For example, it plays an important role in the occurrence and development of human diseases by regulating the expression level and activity of multiple target proteins such as androgen receptors, cell cycle-related proteins, and apoptosis-related proteins. USP14 is abnormally highly expressed in a variety of malignant tumors, and its regulatory mechanisms are complex and diverse, covering the basic characteristics of tumors, such as cell proliferation, apoptosis, inflammation, autophagy, and drug resistance. Meanwhile, high USP14 expression is closely associated with poor prognosis in tumor patients. Especially its potential role in tumor metastasis remains to be explored [9–13].

KPNA2, a constituent of the karyopherin α family, is composed of 529 amino acid residues and plays a vital role as an intermediary in transporting molecules between the nucleus and cytoplasm. Research has shown that KPNA2 plays a role in regulating the transportation of molecules, both from the cytoplasm to the nucleus and vice versa. The expression of KPNA2 has been observed to be aberrant in various malignancies, including prostate cancer, colorectal cancer, and hepatocellular carcinoma [14]. The research conducted by Chen Li and colleagues has also discovered an increased expression of KPNA2 [15], which has been associated with a negative prognosis for GC. Nevertheless, evidence of the potential regulatory mechanisms and specific functions of KPNA2 in gastric cancer metastasis is still lacking [16]. Our findings indicate a significant upregulation of USP14 expression in gastric cancer, which correlates with unfavorable prognosis among patients. Additionally, the suppression of USP14 hampers the migratory and invasive potential of gastric cancer cells. Mechanistically, we found that USP14 interacts with KPNA2 proteins, and USP14 regulates the stability of KPNA2 by deubiquitinating KPNA2, thus regulating the nuclear localization of c-MYC.

## Results

### High USP14 expression is associated with poor prognosis in patients with gastric cancer

**Fig. 1:**
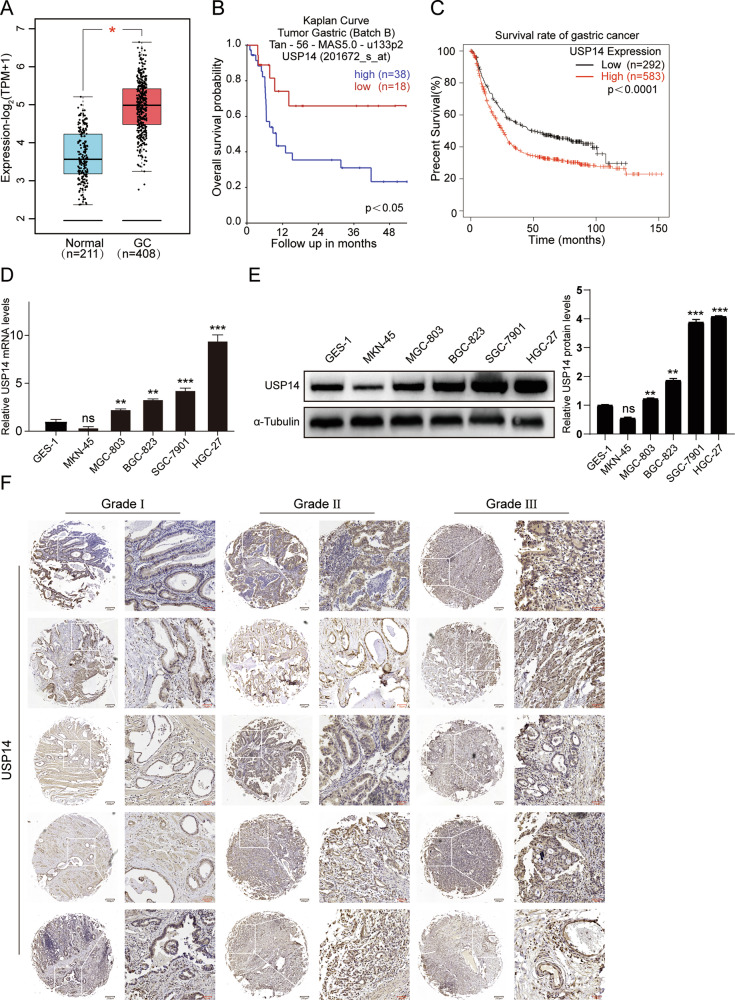
High USP14 expression is associated with poor prognosis in patients with gastric cancer. **A** Box plot of USP14 expression levels in the peritumoral tissues (normal) and GC tumors with log-rank test *P* values < 0.05. **B**, **C** Kaplan–Meier analysis of progression-free survival using data from the R2 database and Kaplan-Meier Plotter database. **D**, **E** qRT-PCR and Western blot assays were used to detect the expression of USP14 in the human normal gastric cell line (GES-1) and GC cell lines (MKN-45, MGC-803, BGC-823, SGC-7901, HGC-27). **F** Immunohistochemical staining analysis showed the expression of USP14 in different stages of gastric cancer tissues. Scale bar(black) = 200 μm. Scale bar(red) = 50 μm. All data are expressed as the mean ± SD. Student’s *t* test was performed to analyze significance. *P < 0.05, **P < 0.01, ***P < 0.001.

To investigate the potential correlation between USP14 expression levels and the prognosis of patients diagnosed with gastric cancer, we first conducted preliminary mining in the database. According to the GEPIA database, a significant upregulation of USP14 expression was observed in gastric cancer tissues compared to normal tissues. (Fig. 1A). The results of two GC datasets also showed that gastric cancer patients with high USP14 expression had poor prognosis (Fig. 1B, C). At the cellular level, we further detected the expression of USP14 in five gastric cancer cell lines MKN-45, MGC-803, BGC-823, SGC-7901, HGC-27, and a normal gastric cell line GES-1 by qRT-PCR. We found that USP14 expression was significantly higher in four gastric cancer cell lines than in normal gastric mucosal cell line (Fig. 1D). Further Western blot analysis also revealed high expression levels of this protein in GC cell lines (Fig. 1E). Finally, the expression of USP14 in tumor tissues of patients with different grades of gastric cancer was detected by immunohistochemical staining. The results showed that USP14 expression was relatively enriched at the invasive edge of the tumor, although positive staining was also detected in other tumor regions (Fig. 1F), and the higher the grade, the higher the USP14 expression level (Fig. S1A). The combined findings suggest a significant upregulation of USP14 in gastric cancer tissues, indicating its potential implication in the development and progression of GC.

### USP14 promotes the proliferation, migration, and invasion of gastric cancer cells

**Fig. 2:**
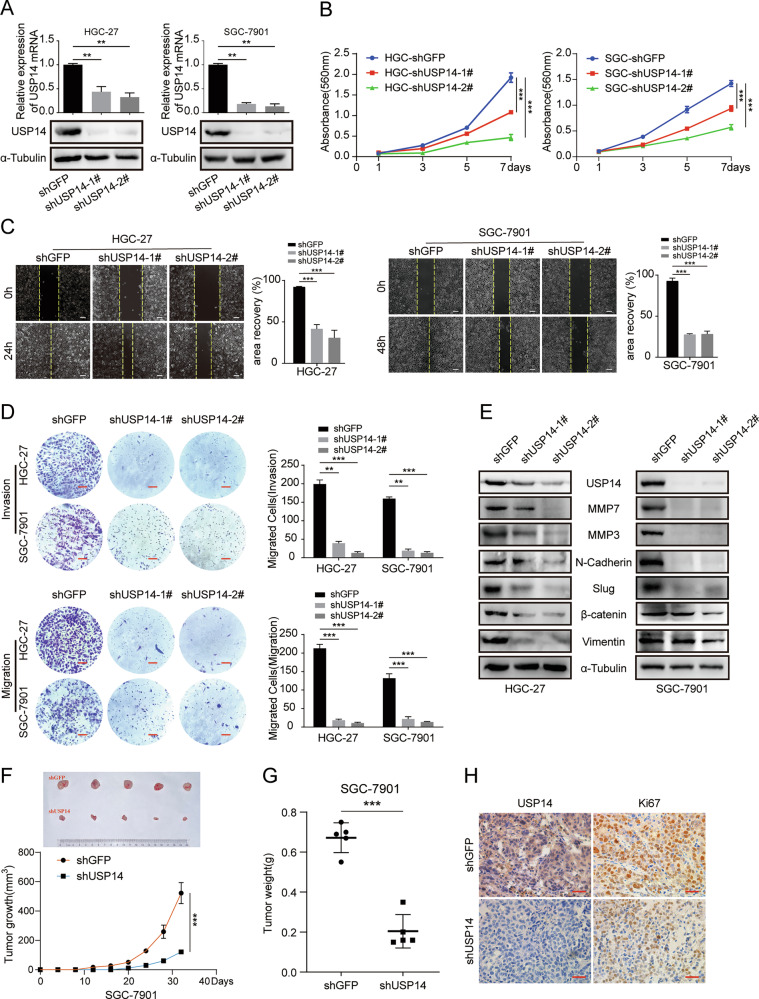
USP14 promotes the proliferation, migration, and invasion of gastric cancer cells. **A** qRT-PCR and Western blot assays were performed to characterize the expression of USP14 in the control and USP14-knockdown HGC-27 and SGC-7901 cells. **B** MTT assay was performed to test the proliferation of the control and USP14-knockdown HGC-27 and SGC-7901 cells. **C** Wound healing assay was performed in USP14-knockdown cells. **D** Migration and invasion assays were performed in USP14-knockdown cells. Scale bar = 50 μm. **E** Western blot assay was performed to characterize the expression of some key metastasis-related proteins in USP14-knockdown cells. **F**, **G** Photographs, growth monitoring, and weights of the indicated xenograft tumors. Data were analyzed using two-tailed Student’s *t* tests. **H** IHC analysis of USP14 and Ki67 expression was carried out in the indicated xenograft tumors. Scale bar = 50 μm. All data were expressed as the mean ± SD. Student’s *t*test was performed to analyze significance. *P < 0.05, **P < 0.01, ***P < 0.001.

To investigate the impact of USP14 on the progression of GC cells, we effectively suppressed the expression of USP14 by employing lentiviruses containing shRNA sequences to treat HGC-27 and SGC-7901 cells (Fig. 2A). The MTT assay was utilized to evaluate the impact of downregulating USP14 expression on the growth of HGC-27 and SGC-7901 cells. The results demonstrated a significant inhibition in the proliferative capacity of gastric cancer cells following USP14 knockdown (Fig. 2B). The plate cloning assay also demonstrated a significant inhibition of cell colony formation in HGC-27 and SGC-7901 cells upon knockdown of USP14 (Fig. S2A). In addition, the addition of USP14-specific inhibitor IU1(IU1 is a small-molecule inhibitor that specifically binds to the activated form of Usp14 in vitro and inhibits its catalytic activity) [17] also inhibited the proliferation of GC cells (Fig. S2B, C). To confirm the crucial role of USP14 in GC metastasis, we performed migration and invasion assays (Fig. 2C, D), which showed that the migration rate of GC cells was significantly reduced upon knockdown of USP14 compared to the control cells. The addition of the specific inhibitor of USP14 IU1 also showed that the migration and invasion ability of gastric cancer cells was inhibited (Fig. S2D, E). Furthermore, the study also examined the presence of certain proteins associated with metastasis. Results from Western blot assays indicated that USP14 knockdown had a significant impact on reducing the expression of proteins linked to proliferation and invasion (Fig. 2E and Fig. S3). The subcutaneous xenograft study demonstrated a significant reduction in growth rate, tumor volume, and weight of SGC-7901 cells with USP14 knockdown compared to control SGC-7901 cells. Moreover, immunohistochemical analysis revealed a decrease in Ki67 expression within tumor tissue sections following USP14 knockdown (Fig. 2F–H). The findings suggest that suppression of USP14 expression hinders the proliferation, motility, infiltration, and progression of gastric cancer cells.

### USP14 recovery rescues the cell proliferation, migration, and invasion of USP14 silenced GC cells

**Fig. 3:**
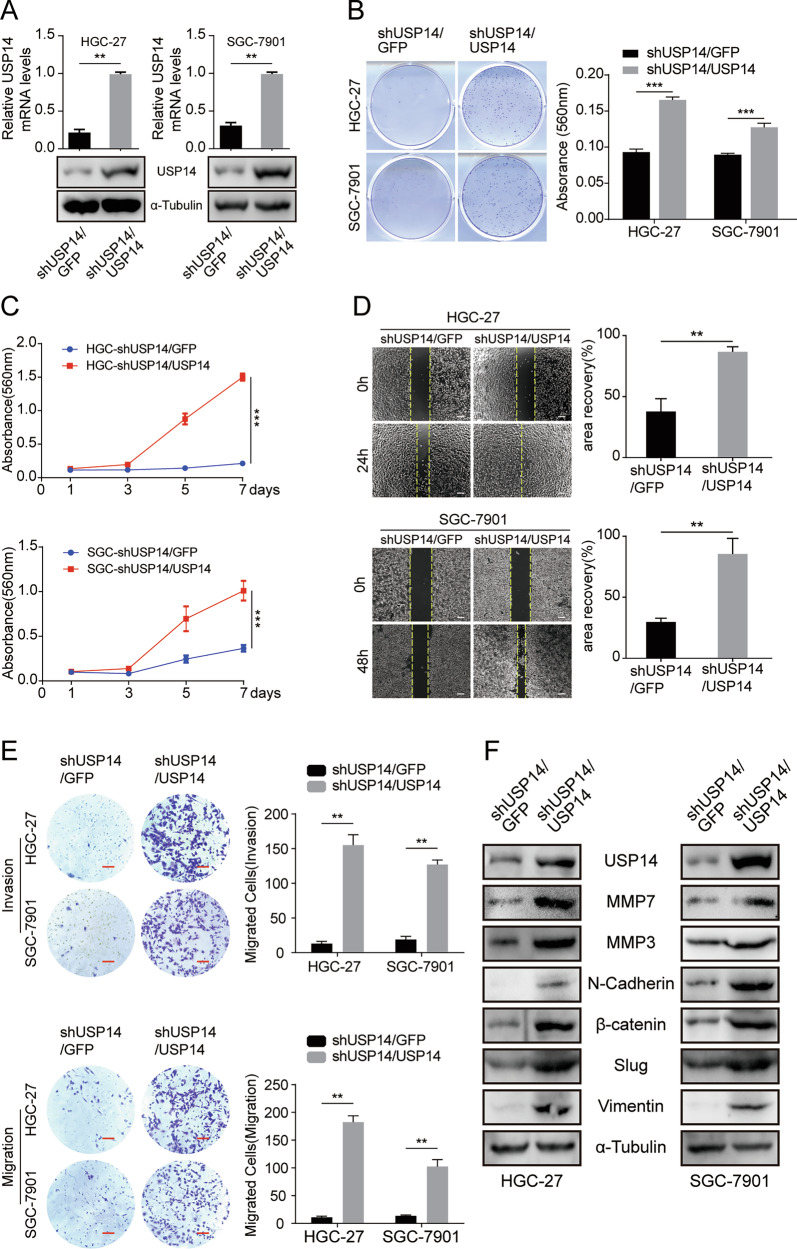
USP14 recovery rescues the cell proliferation, migration, and invasion of USP14-silenced GC cells. **A** qRT-PCR and Western blot assays were used to confirm USP14 expression in USP14-rescued USP14-knockdown HGC-27 and SGC-7901 gastric cancer cells. **B** Plate cloning assays were performed to examine the proliferation of the USP14-rescued USP14-knockdown cells HGC-27 and SGC-7901 cells. **C** Growth curves are shown for the USP14-rescued USP14-knockdown cells. **D** Wound healing assay was performed in USP14-rescued USP14 knockdown cells. **E** Migration and invasion assays were performed with USP14-rescued USP14-knockdown HGC-27 and SGC-7901 cells (left), and the quantification of migratory or invasive cells (right). Cells were stained with crystal violet and counted. Scale bar = 50 μm. **F** Western blot assay was used to detect the protein expression levels of metastasis-related proteins in USP14-rescued USP14-knockdown HGC-27 and SGC-7901 gastric cancer cells.

To further validate the involvement of USP14 in the proliferation and metastasis of gastric cancer (GC) cells, we conducted a transfection experiment using a full-length USP14 sequence against shRNA#1 targeting USP14. The results demonstrated successful recovery of both USP14 protein and messenger RNA (mRNA) expression levels, thereby excluding any potential off-target effects (Fig. 3A). Recovery of cell growth and proliferation was observed upon USP14 expression, as demonstrated by plate cloning (Fig. 3B) and MTT (Fig. 3C) assays. Furthermore, the migratory and invasive abilities of shUSP14 cells were significantly restored when USP14 expression was recovered(Fig. 3D, E). In addition, the levels of various proteins associated with metastasis were assessed, and it was observed that the restoration of USP14 resulted in notable enhancements in the expression of MMP7, N-cadherin, and vimentin. (Fig. 3F and Fig. S4). Collectively, these results indicate that the role of USP14 is indispensable in facilitating the proliferation, migration, and invasion of gastric cancer cells.

### USP14 interacts with KPNA2 and governs KPNA2 stability

**Fig. 4:**
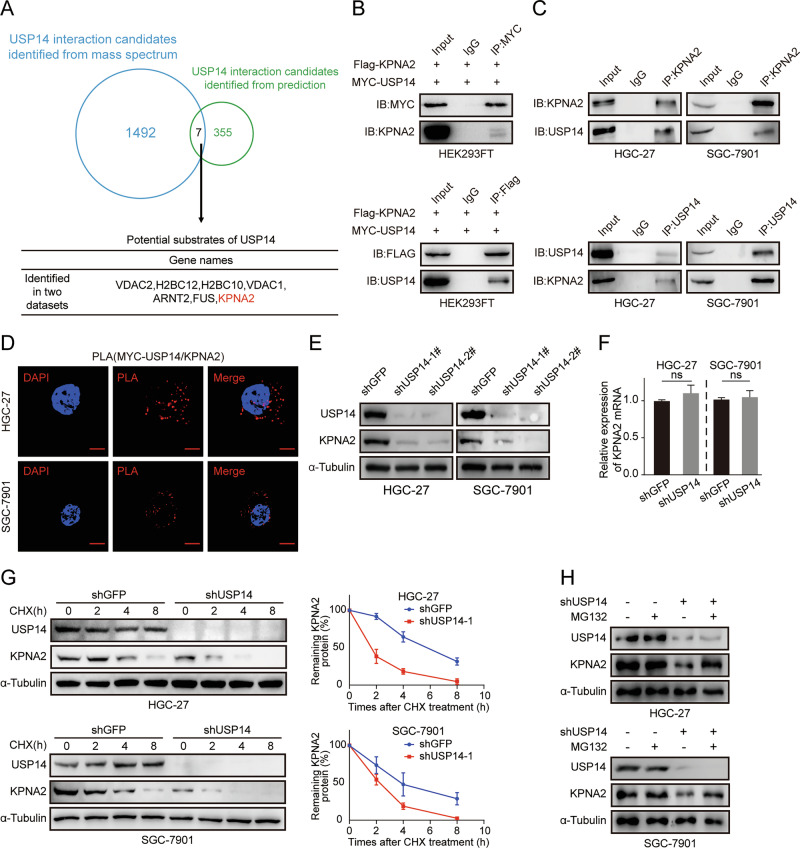
USP14 interacts with KPNA2 and governs KPNA2 stability. **A** Venn diagram showing the number of USP14 interaction candidates identified from the prediction (green), USP14 interaction candidates identified in the Co-IP process (blue), and overlapping proteins in the datasets. The chart below shows the potential substrates of the overlapped proteins. **B** HEK293FT cells were co-transfected with MYC-USP14 and Flag-KPNA2 plasmids and analyzed after co-precipitation with anti-Flag or anti-MYC antibody immunoprecipitation. **C** HGC-27 and SGC-7901 cells were immunoprecipitated with anti-USP14 or anti-KPNA2 antibodies and analyzed. **D** PLA assay was performed after MYC-USP14 expression in HGC-27 and SGC7901 cells using anti-MYC and anti-KPNA2 antibodies. Scale bar, 2 μm. **E** Western blot assay was used to detect the expression level of KPNA2 in gastric cancer cells after USP14 knockdown. **F** qRT-PCR assay was used to analyze the expression level of KPNA2 in gastric cancer cells after USP14 knockdown. **G** The KPNA2 turnover rate in HGC-27 and SGC-7901 cells with USP14 knockdown. **H** Cell lysates were prepared from knocked-down USP14 cells that had been previously treated with or without MG132 for 8 h.

To investigate the regulatory mechanism of USP14, we performed Co-IP experiments and mass spectrometry to identify its interaction proteins. Subsequently, by utilizing the UbiBrowser 2.0 network database for prediction and intersection analysis, we obtained a gene list that includes the known USP14 substrates HSC70, IDO1, and CXCR4 [18–20]. Finally, through preliminary experiments, we focused on KPNA2, a member of the nuclear transporter family that can regulate the nuclear translocation of various proteins via its nuclear-cytoplasmic transport function. (Fig. 4A). Previous studies have indicated a correlation between KPNA2 and unfavorable outcomes in patients with gastric cancer. IHC, qRT-PCR and Western blot assays showed that KPNA2 was highly expressed in gastric cancer cells; GEPIA2 database also showed that KPNA2 was highly expressed in gastric cancer(Fig. S5 and Fig. S6A–C). After obtaining KPNA2 stably down-regulated cell lines (Fig. S6D), we performed MTT, Wound-healing, and Transwell assays, and detected the expression levels of proliferation, migration, and invasion-related proteins (Fig. S6E–H). These results demonstrated that the knockdown of KPNA2 inhibited the proliferation, migration, and invasion of gastric cancer cells. This was consistent with the changes after USP14 knockdown, Next, we restored KPNA2 expression after knocking down USP14 in gastric cancer cells, performed MTT, Wound-healing, and Transwell assays, and detected the expression levels of proliferation, migration, and invasion proteins (Fig. S7A–D). These results indicated that the knockdown of USP14 followed by restoration of KPNA2 expression partially restored gastric cancer cell proliferation, migration, and invasion ability. We co-transfected MYC-USP14 plasmid and Flag-KPNA2 plasmid into HEK293FT cells, followed by co-immunoprecipitation using MYC and Flag Tag antibodies to detect their interaction. The results demonstrated a reciprocal interaction between exogenous USP14 and KPNA2. (Fig. 4B). The same co-immunoprecipitation assay was used to examine the interaction of endogenous USP14 to KPNA2 in gastric cancer cells HGC-27 and SGC-7901, and the results showed that USP14 interacted with KPNA2. (Fig. 4C). Furthermore, we conducted a proximity ligation assay (PLA) by utilizing antibodies anti-KPNA2 and MYC in HGC-27 and SGC-7901 cells that were transiently transfected with USP14 protein tagged with MYC (Fig. 4D), which further verified the interaction between USP14 and KPNA2. The Western blot assays revealed a significant decrease in the protein level of KPNA2 in gastric cancer cells with USP14 knockdown (Fig. 4E). However, there was no notable reduction observed in the mRNA level of KPNA2 (Fig. 4F), suggesting that posttranscriptional regulation might be involved in the modulation of KPNA2 expression by USP14. Then, we found that overexpression of USP14 decreased the turnover rate of KPNA2 in gastric cancer cells by using the de novo protein synthesis inhibitor CHX(cycloheximide) (Fig. 4G). Furthermore, we found that the decrease in KPNA2 protein expression in USP14 knockdown gastric cancer cells was rescued by using the proteasome inhibitor MG132 (Fig. 4H). In brief, the findings of this study suggest that there is an interaction between USP14 and KPNA2, leading to the regulation of KPNA2 stability through inhibition of its degradation. Additionally, it appears that KPNA2-mediated migration and invasion in gastric cancer cells are under the control of USP14.

### USP14 promotes KPNA2 deubiquitination

**Fig. 5:**
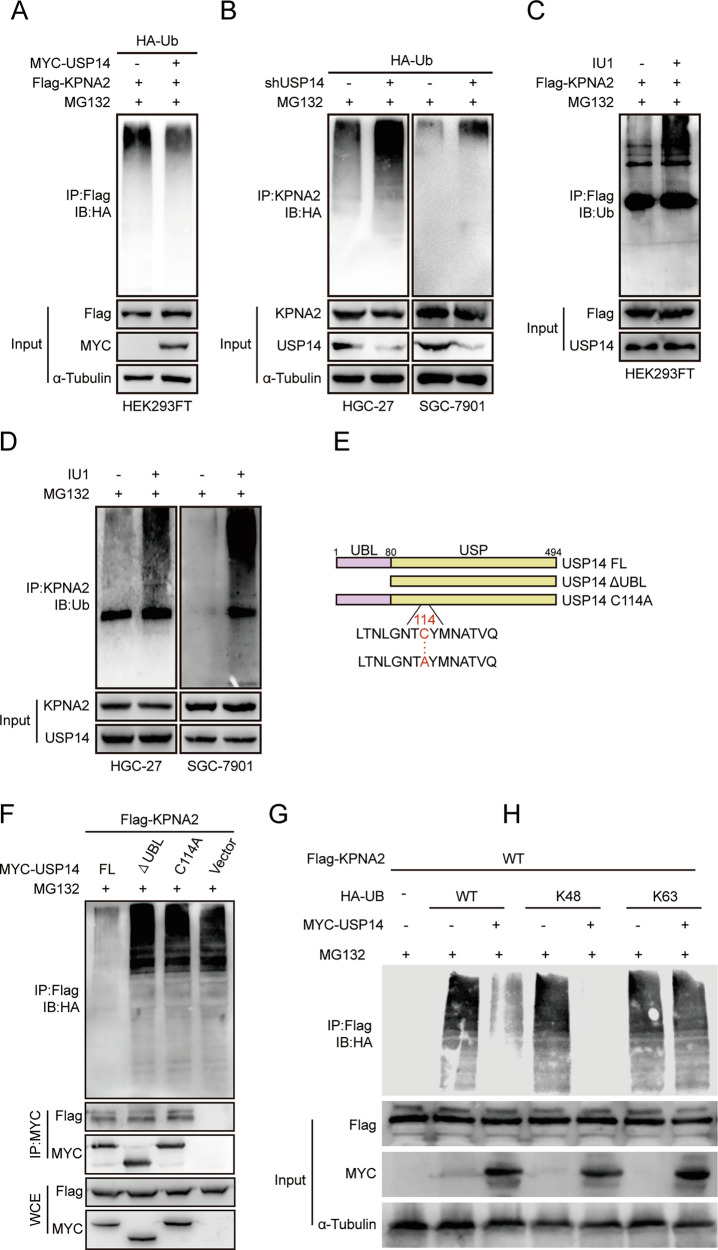
USP14 promotes KPNA2 deubiquitination. **A** HEK293FT cells were transfected with HA-Ubiquitin, Flag-KPNA2, and MYC-USP14 as indicated. Poly-ubiquitination of KPNA2 was then examined by immunoprecipitation with Protein A + G agarose and analyzed. Cells were treated with MG132 (10 μM) for 8 h before being harvested. **B** USP14-knockdown and control HGC-27 and SGC-7901 cells were treated with MG132 (10 μM) for 8 h before being harvested. **C** HEK293FT cells were transfected with Flag-KPNA2 as indicated, treated with IU1 (50 μM) for 24 h, and then treated with MG132 (10 μM) for 8 h before being harvested. Lysates were immunoprecipitated with anti-KPNA2 antibody and analyzed. **D** HGC-27 and SGC-7901 cells treated with IU1 (50 μM) for 24 h were treated with MG132 (10 μM) for 8 h before being harvested. Lysates were immunoprecipitated with anti-KPNA2 antibody and analyzed. **E**, **F** HEK293FT cells were transfected with Flag-KPNA2 and indicated constructs, treated with MG132 (10 μM) for 8 h before being harvested. Lysates were immunoprecipitated with anti-Flag antibody and analyzed. **G**, **H** HEK293FT cells were transfected with HA-K48-Ub, HA-K63-Ub, and Flag-KPNA2 with or without MYC-USP14 as indicated and cultured for 48 h. Cells were immunoprecipitated with anti-Flag antibody and analyzed.

USP14 is an important member of the deubiquitinating enzyme family of USP. Based on the above research, we speculated that USP14 exhibits deubiquitinating enzyme activity to regulate the ubiquitination level of KPNA2, regulates the degradation of KPNA2, and then affects the proliferation, migration, and invasion of gastric cancer cells. We overexpressed the Flag-tagged KPNA2 plasmid in HEK293FT cells and performed ubiquitination assays with overexpression of the MYC-tagged USP14 plasmid as a variable. The results showed that the ubiquitination level of KPNA2 was reduced after USP14 overexpression (Fig. 5A). In gastric cancer cells, we found that the ubiquitination of KPNA2 was significantly enhanced after USP14 knockdown (Fig. 5B). The results of experiments with the addition of a specific inhibitor of USP14, IU1, also confirmed that USP14 inhibition leads to increased ubiquitination of KPNA2 (Fig. 5C, D). An experiment was conducted to determine which domain(s) of USP14 mediated its interaction with KPNA2. Two mutants were constructed: USP14 C114A, which had an active site mutation, and USP14 lacking the ubiquitin-like domain (UBL) 18, and verify whether it affects KPNA2 ubiquitination (Fig. 5E, F) The results showed that the catalytically inactive mutant of USP14 failed to reduce KPNA2 ubiquitination, whereas the wild-type USP14 significantly decreased it, indicating that USP14-mediated deubiquitination of KPNA2 depends on its catalytic activity. The overexpression of USP14 significantly reduced K48 ubiquitination levels, while no significant change was observed in K63 ubiquitination levels of KPNA2 (Fig. 5G, H).

In summary, these results suggested that KPNA2 can bind to USP14 in its UBL domain. In addition, USP14 deubiquitinated KPNA2 by removing the K48 polyubiquitination chains from KPNA2.

### USP14 regulates c-MYC nuclear transport and expression through KPNA2

**Fig. 6:**
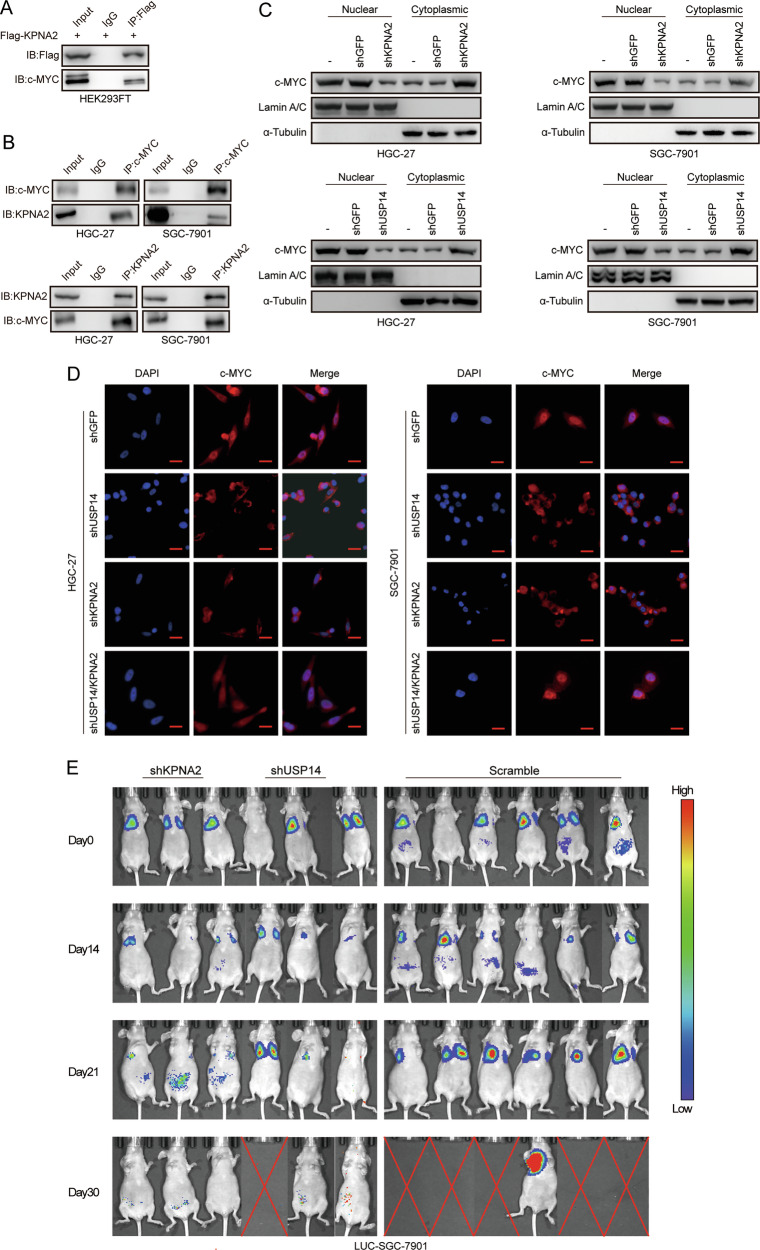
USP14 regulates c-MYC nuclear transport and expression through KPNA2. **A** HEK293FT cells were transfected with Flag-KPNA2 plasmids and analyzed after co-precipitation with anti-Flag antibody immunoprecipitation. **B** HGC-27 and SGC-7901 cells were immunoprecipitated with anti-KPNA2 or anti-c-MYC antibodies and analyzed. **C**, **D** The subcellular distribution of c-MYC in the USP14-shRNA, KPNA2-shRNA, and control transfected cells by nuclear/cytosol fractionation and immunofluorescence was shown. α-Tubulin was used as the cytoplasmic control and Lamin A/C as the nuclear control. **E** In vivo imaging experiments were conducted to assess the metastatic behavior of gastric cancer SGC-7901 cells with USP14 and KPNA2 interference in mice.

To further explore the specific mechanism by which USP14 regulates gastric cancer cell migration and invasion through KPNA2, we verified the interaction of KPNA2 with c-MYC by Co-IP experiments in HEK293FT and gastric cancer cells, respectively (Fig. 6A, B). Then, we performed nucleoplasmic separation experiments on gastric cancer cells with USP14 and KPNA2 knockdown, respectively. Knockdown of USP14 and KPNA2 reduced the expression of c-MYC in the nucleus (Fig. 6C), and immunofluorescence experiments also showed that the fluorescence signal in the nucleus was significantly reduced after knocking down USP14 and KPNA2. However, the restoration of KPNA2 expression restored c-MYC nuclear localization in the nucleus (Fig. 6D). After transfecting LUC-SGC-7901 cells with USP14 and KPNA2 interference, and then injecting them into mice via the tail vein, it was found that their metastatic ability was significantly reduced compared to the control group (Fig. 6E). These results suggested that USP14 regulates the migration and invasion of gastric cancer cells by regulating KPNA2 and regulating the nuclear translocation of c-MYC.

## Discussion

Gastric cancer (GC) is a prevalent form of cancer globally, known for its frequent recurrences and extensive infiltration into the adjacent healthy tissue and blood vessel formation [21]. Despite the worldwide decrease in occurrence throughout the last century, gastric cancer (GC) continues to be a major cause of death globally. Hence, it is imperative to explore the molecular mechanisms that underlie GC.

USP14, a member of the ubiquitin-specific protease family, has been observed to display increased expression in certain types of human cancers and is involved in crucial functions related to tumor formation and advancement [11–13]. In this study, functionally we identified an important role of USP14 in promoting gastric cancer proliferation and metastasis, which was also supported by previous findings in different cell lines, suggesting that USP14 may be a potential therapeutic target in gastric cancer. Mechanistically, we found that USP14 deubiquitinated KPNA2 and regulated c-MYC nuclear localization through KPNA2.

As a member of the nuclear transport protein family, KPNA2 regulates the nuclear translocation of a variety of proteins through its nucleoplasmic transport function and participates in a variety of life activities such as proliferation, apoptosis, migration, invasion, transcriptional regulation, immune response, and virus infection [14, 22, 23]. Recent studies have shown that the expression of KPNA2 is up-regulated in a variety of tumor tissues, including gastric cancer [24], and the abnormal expression of KPNA2 is associated with poor prognosis of patients [25]. There are currently some reports on mechanisms of action of KPNA2 in tumors. For example, the knockdown of KPNA2 in non-small cell lung cancer resulted in the subcellular redistribution of E2F [26]. Similarly, the knockdown of KPNA2 downregulated c-MYC and reduced its transcriptional activity [27]. These results suggest that KPNA2 plays a vital role in tumor formation and progression.

The regulation of various transcriptional programs and the crucial role in the progression of numerous human cancers are attributed to the transcriptional activator c-MYC. Under normal physiological conditions, multiple cellular mechanisms strictly govern the expression of c-MYC [28]. While the normal growth and proliferation of cells necessitate c-MYC, the abnormal activation or excessive expression of c-MYC is linked to the onset and progression of a majority of human malignancies [29]. The impact of the c-MYC gene on controlling the proliferation, migration, and invasion of GC cells has been examined [30]. In addition, it has been reported that KPNA2 can regulate the nuclear translocation of c-MYC in breast cancer [31], and KPNA2 can regulate c-MYC expression in the nucleus by mediating the nuclear translocation of E2F1 in glioma [32].

In our investigation, it was observed that the depletion of USP14 and KPNA2 resulted in a reduction in the nuclear abundance of c-MYC. The reintroduction of KPNA2 expression led to a restoration of c-MYC localization within the nucleus. It is worth noting that, according to previous literature and multiple ubiquitination-site prediction databases (e.g., PhosphoSitePlus, UbiBrowser, and UbPred), KPNA2 harbors numerous potential ubiquitination sites distributed across different structural domains [33, 34]. Generating mutants for all these lysine residues to prevent ubiquitination would be technically challenging, and extensive mutagenesis could inadvertently alter the intrinsic structure or conformation of KPNA2. Such structural perturbations may introduce additional variables, making it difficult to draw definitive conclusions regarding the site-specific regulation by USP14. In the present study, although we did not perform ubiquitination-site mutagenesis, our combined evidence from K48/K63 chain-specific ubiquitination assays, CHX-mediated degradation assays, and MG132 rescue experiments strongly supports the conclusion that USP14 stabilizes KPNA2 through K48-linked deubiquitination. The fact that there are still unresolved issues deserving further investigation is worth noting. For example, several questions remain unanswered.

Does USP14 promote cell apoptosis? What is the specific ubiquitination site of KPNA2? Which specific amino acids mediate the binding between USP14 and KPNA2? What is the precise mechanism by which USP14 regulates KPNA2 deubiquitination? Are there additional genes involved in regulating this ubiquitination process? Clinically, whether targeting the USP14 gene can be used in combination with other clinical first-line drugs such as 5-fluorouracil remains to be explored. Further investigation into these matters will contribute to a comprehensive understanding of the increased stability of KPNA2 resulting from the elevated expression of USP14, thereby presenting a more valuable prospective focus for targeted therapy in GC. These aspects present intriguing subjects that merit additional exploration.

Taken together, this study showed that USP14 promotes the proliferation, migration, invasion, and tumor growth of gastric cancer cells. Furthermore, we found that USP14 plays a regulatory role in gastric cancer cells by regulating KPNA2-mediated c-MYC nuclear translocation through deubiquitination. These findings provide new insights into the biological function of USP14 and suggest that USP14 can serve as a promising target for the treatment of gastric cancer.

## Materials and methods

### Cell lines, drugs, reagents and antibodies

All human GC cell lines (BGC-823, HGC-27, MGC-803, MKN-45, and SGC-7901), Human normal gastric cell line (GES-1), and human embryonic renal cell line HEK293FT were obtained from the American Type Culture Collection (ATCC, Beijing, China). All cell lines were tested mycoplasma-negative. MG132 and CHX were obtained from Sigma (Shanghai, China). IU1 was purchased from MedChemExpress (Shanghai, China, Cat#HY-13817). This inhibitor has been reported to selectively inhibit USP14 activity over other deubiquitinating enzymes. Anti-KPNA2 (Cat# 10819-1-AP), anti-MMP2 (Cat# 10373-2-AP), anti-MMP3 (Cat# 17873-1-AP), anti-MMP7 (Cat# 10374-2-AP), anti-Slug (Cat# 12129-1-AP), anti-E-Cadherin (Cat# 20874-1-AP), anti-N-cadherin (Cat# 22018-1-AP), anti-α-Tubulin (Cat# 11224-1-AP), anti-HA (Cat# 51064-2-AP), anti-Ub (Cat# 10201-2-AP) antibodies were purchased from Proteintech (Wuhan, China). Anti-MYC (Cat#2276), anti-Flag (Cat#14793), anti-c-MYC (Cat#13987), anti-Vimentin (Cat#5741), anti-USP14 (Cat#11931), and anti-β-catenin (Cat#9562) antibodies were obtained from Cell Signaling Technology (Shanghai, China). Anti-Ki67(Cat#550609) antibody was purchased from BD Biosciences. All antibodies were diluted according to the manufacturer’ s instructions.

### Transfection and infection experiments and plasmids

Small-hairpin shRNAs for USP14 and KPNA2 and a negative control shRNA (shGFP) were obtained from Sangon Biotech. (Shanghai, China) and were inserted into the pLKO.1 vector. The ubiquitination plasmid that contained an HA tag and recombinant plasmids containing full-length human USP14 and KPNA2 cDNA cloned into the PCDH-CMV-MCS-EF1-Hygro vector were purchased from Youbao Company (Changsha, China). The ubiquitin mutant plasmids (K48, K63) containing the HA tag were also purchased from Youbao Company (Changsha, China). For transfection and infection experiments, the target plasmids and packaging plasmids were transfected into HEK293FT cells by using the transfection reagent Lipofectamine 2000 (Invitrogen, Carlsbad, CA, USA). Lentiviruses were collected 48 h later and used to infect GC cells twice, 24 h per infection. The infected cells were screened by treatment with puromycin for 36 h, and the surviving cells were frozen and stored in liquid nitrogen for subsequent experiments. For shUSP14 and shKPNA2 without sequence number indicated, interference sequence number 2 was the default. All the primers for the shRNA sequences are given in Table 1.

**Table 1:** Sequence of the shRNA primers.

shUSP14#1-F	CCGGCCCAAGATTCAGCAGTCAGATCTCGAGA TCTGACTGCTGAATCTTGGGTTTTTG
shUSP14#1-R	AATTCAAAAACCCAAGATTCAGCAGTCAGATC TCGAGATCTGACTGCTGAATCTTGGG
shUSP14#2-F	CCGGCGCAGAGTTGAAATAATGGAACTCGAGT TCCATTATTTCAACTCTGCGTTTTTG
shUSP14#2-R	AATTCAAAAACGCAGAGTTGAAATAATGGAAC TCGAGTTCCATTATTTCAACTCTGCG
shKPNA2#1-F	AATTCAAAAAGCTGGTTTGATTCCGAAATTTCT CGAGAAATTTCGGAATCAAACCAGC
shKPNA2#1-R	AATTCAAAAAGCTGGTTTGATTCCGAAATTTCT CGAGAAATTTCGGAATCAAACCAGC
shKPNA2#2-F	CCGGCCTGGACACTTTCTAATCTTTCTCGAGAA AGATTAGAAAGTGTCCAGGTTTTTG
shKPNA2#2-R	AATTCAAAAACCTGGACACTTTCTAATCTTTCT CGAGAAAGATTAGAAAGTGTCCAGG

The shRNA sequences are listed below.

### Immunohistochemistry staining

Paraffin-embedded tumors were cut into 5 mm thick slices, and then the paraffin sections were dewaxed and hydrated. Then, paraffin slices were placed in citrate buffer (pH 6.0) and heated in a microwave oven to 95 °C for 20 min to facilitate antigen retrieval. Then, endogenous peroxidase activity was quenched, followed by blocking with normal goat serum. Then, USP14 and Ki67 antibodies were diluted with BSA according to the manufacturer’s instructions, and the antibodies were added to the paraffin sections and incubated overnight at 4 °C. Then, a horseradish peroxidase-linked secondary antibody was added and incubated with the sections, which was followed by the addition of DBA reagent. The results were observed under a microscope before counterstaining with hematoxylin.

### Quantitative and reverse transcription PCR

Total RNA was extracted from cells using TRIzol reagent. Then, 2 µg of RNA was reverse-transcribed into cDNA. The normalized expression control was based on the glyceraldehyde-3-phosphate dehydrogenase value. Finally, mRNA expression was determined as the CT value. All quantitative primers are given in Table 2.

**Table 2:** Sequence of the RT-PCR primers.

USP14-forward	CCGGCCCAAGATTCAGCAGTCAGATCTCGAGATC TGACTGCTGAATCTTGGGTTTTTG
USP14-reverse	AATTCAAAAACCCAAGATTCAGCAGTCAGATCTC GAGATCTGACTGCTGAATCTTGGG
KPNA2-forward	CCGGCGCAGAGTTGAAATAATGGAACTCGAGTT CCATTATTTCAACTCTGCGTTTTTG
KPNA2-reverse	AATTCAAAAACGCAGAGTTGAAATAATGGAACT CGAGTTCCATTATTTCAACTCTGCG
GAPDH-forward	CCGGGCTGGTTTGATTCCGAAATTTCTCGAGAAA TTTCGGAATCAAACCAGCTTTTTG
GAPDH-reverse	AATTCAAAAAGCTGGTTTGATTCCGAAATTTCTC GAGAAATTTCGGAATCAAACCAGC

Primer pairs for real-time PCR assays.

RIPA was used for cell lysis to extract protein from the cells, and then, proteins were denatured and separated. Proteins of different molecular weights were separated by SDS-polyacrylamide gel electrophoresis and electrotransferred to polyvinylidene difluoride membranes. The membrane is sealed with skimmed milk to detect the proteins, and BSA to detect the phosphorylated proteins, and the membrane sections were incubated sequentially with primary antibodies, and then, secondary antibodies. The membranes were exposed to ECL Reagent (Cell Signaling) and visualized by Western blot analysis detection system (Thermo Fisher, Shanghai, China).

### Plate cloning

To clone plates, a six-well plate was seeded with 1 × 10^3 cells on coverslips. After two weeks, crystal violet staining solution was used to stain the cells and they were scanned using a scanner. The cells were then decolorized with absolute ethanol and shaken sufficiently before measuring absorbance at 560 nm. A graph was plotted based on the absorbance data obtained.

### MTT assay

1 × 10^3 cells were seeded in each well of a 96-well plate and cultured for seven days. MTT reagent was added to the well and incubated at 37 degrees for two hours. After all the liquid in the well was discarded, DMSO (CAS:67-68-5, Sangon Biotech) reagent was added to each well and incubated at 37 degrees for 10 min. The absorbance value was detected and plotted at 560 nm after the plate was shaken by the microplate reader. All experiments were independently performed three times.

### Wound-healing assays

The cells were grown until they reached 80% confluence and subjected to 24 h of nutrient deprivation (0.1% FBS). A single wound was inflicted using a micropipette tip. Following rinsing, the cells were exposed to a medium containing 3% FBS at 37 °C to facilitate cell migration.

### Transwell migration and invasion assay

Migration and invasion assay were conducted using chambers comprising Transwell membrane filter inserts (Cat # 3422, Corning Costar). Briefly, a total of 5 × 10^4 cells were seeded into each well of the 24-well Transwell chamber (with an 8 μm pore size) for the migration assay. For the invasion assay, cells were seeded into Matrigel-coated chambers. The seeding was performed in complete medium supplemented with 10% FBS. Non-penetrating cells on the filter were removed by wiping, and the cells on the lower surface of the filter were stained using a solution containing 0.4% crystal violet dye. The number of migrating or invading cells was determined by counting under a light microscope from three fields within one chamber per sample (mean ± SE).

### Xenograft assay

The Animal Protection and Utilization Committee of Southwest University approved conducting animal experiments. All experimental procedures adhered to the Guidelines for Use (Ministry of Science and Technology, China, 2006). Female NOD/SCID mice were procured and housed in a specific pathogen-free (SPF) room with controlled temperature and humidity at four weeks old. Each mouse received a slow injection of 1 × 10^5 human GC cells (SGC-7901) stably transfected with shGFP or shUSP14#2 into one side of their armpit. Upon completion of the experiment, the tumors were excised, processed, and subjected to analysis. Randomization was employed along with single blinding during measurements. Finally, the tumors were collected and photographed for subsequent immunohistochemical staining following previously established protocols.

### Immunoprecipitation

To begin, HEK293FT cells or gastric cancer cells were transfected with a specific plasmid and subsequently lysed IP lysis buffer. Following this, proteins were extracted from the cells. The target protein was then incubated overnight at 4 °C with a specific proportion of primary antibody. Subsequently, a solution containing 50 µl protein A + G agarose beads was added to the protein and antibody mixture and incubated for 4 h at 4 °C. After incubation, the target proteins became attached to the beads, which were washed using precooled PBS buffer to eliminate impurities. To prepare for further analysis, the protein samples were mixed with 40 µl of 1×loading buffer and subjected to heat-denaturation. Next, these samples underwent separation through SDS polyacrylamide gel electrophoresis before being electrotransferred onto polyvinylidene difluoride membranes. These membranes were left to incubate overnight at 4 °C with primary antibodies followed by an additional 2-hour incubation period with secondary antibodies. Finally, exposure and analysis of the membranes took place utilizing a Chemiscope 6000 imaging system.

### Proximity ligation assay (PLA)

The lentivirus-infected cells were sequentially subjected to puromycin selection, paraformaldehyde fixation, goat serum blocking, and overnight incubation with MYC and KPNA2 antibodies. Subsequently, The cells underwent exposure to a secondary antibody that was labeled with Alexa Fluor 594 (1:1000), along with utilization of a PLA assay Kit provided by Sigma-Aldrich®. Finally, confocal fluorescence microscopy (Olympus Fv1000, Japan) was employed for visualization and photography of the cells.

### Turnover assay

The cells infected with the virus were puromycin for a duration of 48 h, followed by treatment with CHX at a concentration of 50 µg/ml. Subsequently, the cells were gathered, lysed, and subjected to Western blot analysis.

### Ubiquitination assay

To conduct the in vivo ubiquitination assay, designated plasmids were transfected into either HEK293FT cells or gastric cancer cells using a co-transfection approach. After 48 h of transfection, the cells were exposed to MG132, a proteasome inhibitor, at a concentration of 50 μg/ml for a duration of 8 h. Subsequently, Cell Lysis Buffer (Sigma) was employed to lyse the cells for Western blot and IP analysis following an identical protocol utilized for Co-immunoprecipitation.

### Subcellular fractionation

The cells were harvested and washed twice with PBS. Subcellular fractionation was then performed using the Nuclear and Cytoplasmic Protein kit (Beyotime Biotechnology, Wuhan, China) according to the manufacturer’s instructions. The effectiveness of fractionation was assessed through immunoblotting analysis utilizing anti-Lamin A/C antibody (Proteintech, Wuhan, China) as a marker for nuclear proteins and anti-α-Tubulin antibody (Proteintech, Wuhan, China) as a marker for cytosolic proteins.

### Immunofluorescence assay

An immunofluorescence analysis was conducted to identify the presence of c-MYC in GC cells. In brief, cells were gathered, fixed, and coated. Following blocking, the cells were exposed to an anti-c-MYC antibody (1:500) overnight at 4 °C. Subsequently, the cells were treated with a secondary antibody labeled with Alexa Fluor 594 (1:2000). The nuclear staining was performed using Hoechst 33342 (1:2000) subsequently. Under a fluorescence microscope, images of the cells were captured.

### Patient data analysis

The gene expression data were obtained from the GEPIA2 database (http://gepia2.cancer-pku.cn), while the prognostic data were acquired from the R2:Genome Analysis and Visualization Platform (https://hgserver1.amc.nl/cgi-bin/r2/main.cgi). Additionally, we utilized Kaplan-Meier Plotter database (www.kmplot.com) to determine the critical value for segregating the high-expression group and low-expression group, based on algorithms provided by these databases.

### Statistical analysis

The experiments were conducted in triplicate, and the figure captions provide information on statistical parameters such as sample size and significance analysis. A two-tailed Student’s *t* test was employed to determine significance at a 95% confidence level, assuming a normal distribution with slightly different yet comparable standard deviations. The quantitative data is presented as mean ± s.d., and statistical significance was considered for *P* values less than 0.05.

## Supplementary information

SUPPLEMENTAL MATERIAL

WB RAW DATA

mass spectrometry results

## Data Availability

All data are available in the main text or the supplementary materials.
