# Supplementary material for: The deubiquitination enzyme USP14 promotes the tumourigenesis of gastric cancer by enhancing c-MYC nuclear translocation through deubiquitination of KPNA2
Source: Cell Death Dis. 2025 Oct 21;16(1):737. doi: 10.1038/s41419-025-08065-2 (PMC12540732; doi:10.1038/s41419-025-08065-2)
Supplement: Supplementary file 1 — SUPPLEMENTAL MATERIAL [file 41419_2025_8065_MOESM1_ESM.pdf]

Supplementary Materials for

The deubiquitination enzyme USP14 regulates the tumourigenesis of gastric cancer by regulating c-MYC nuclear translocation through deubiquitination of KPNA2

Jia Li *et al.*

*Corresponding author. Email: erhuzhao@126.com

Fig.S1 High USP14 expression is associated with poor prognosis in patients with gastric cancer. (A) IHC for USP14 expression levels in different stages of gastric cancer tissues.

A

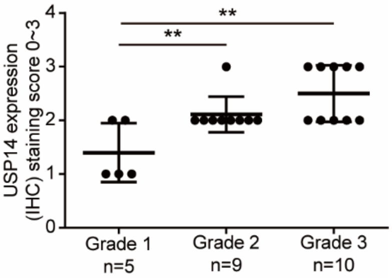

Fig.S2 USP14 promotes the proliferation, migration, and invasion of gastric cancer cells.

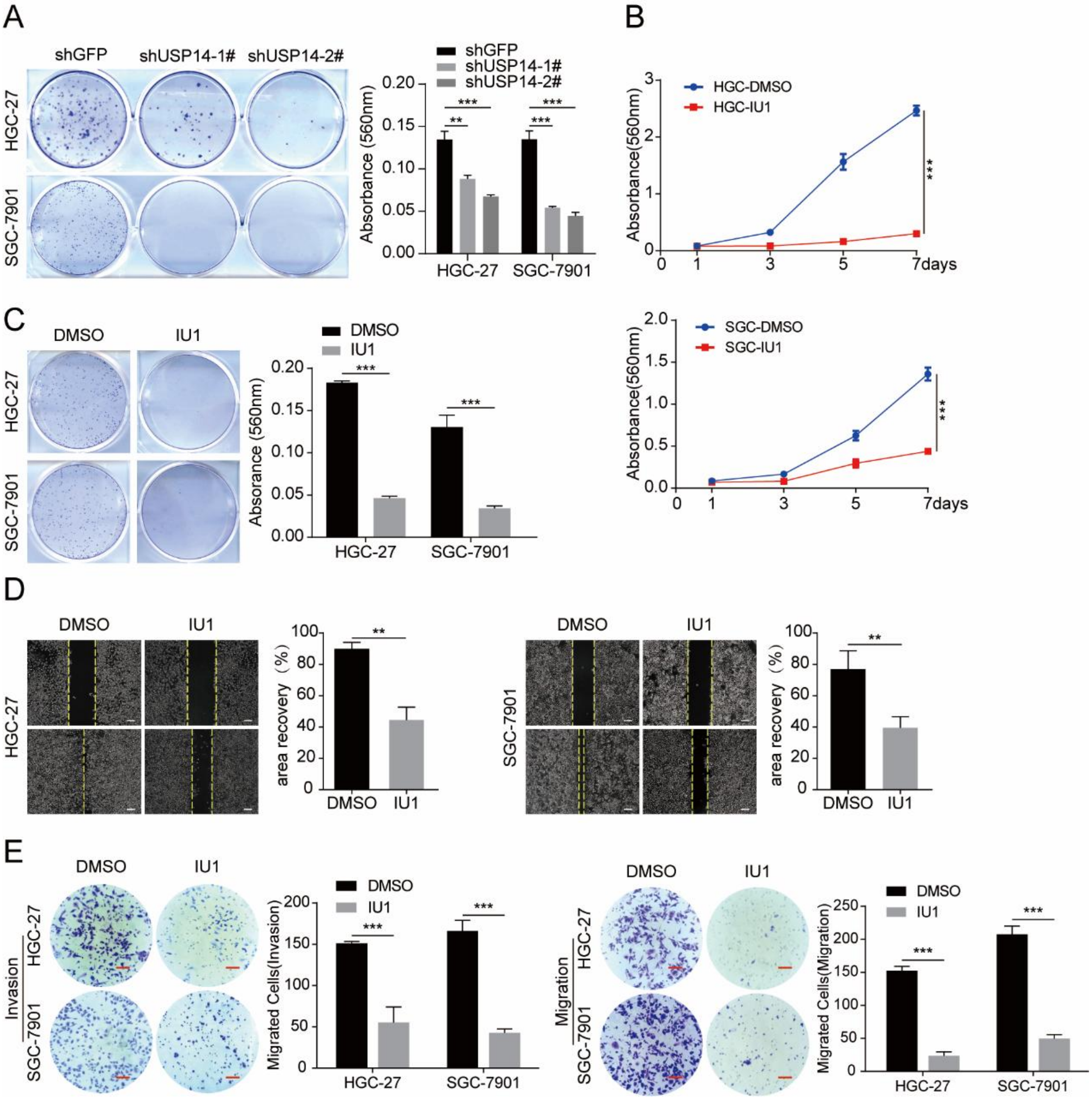

(A) Plate cloning assay was performed to examine the proliferation of the control and USP14-knockdown HGC-27 and SGC-7901 cells.

(B-E) MTT assay Plate cloning assay Wound-healing assay Transwell migration and invasion assays were performed to examine the proliferation ability of HGC-27 and SGC-7901 cells after adding USP14 specific inhibitor IU1 (DMSO as control).

Fig.S3 USP14 knockdown had a significant impact on reducing the expression of proteins linked to proliferation and invasion.

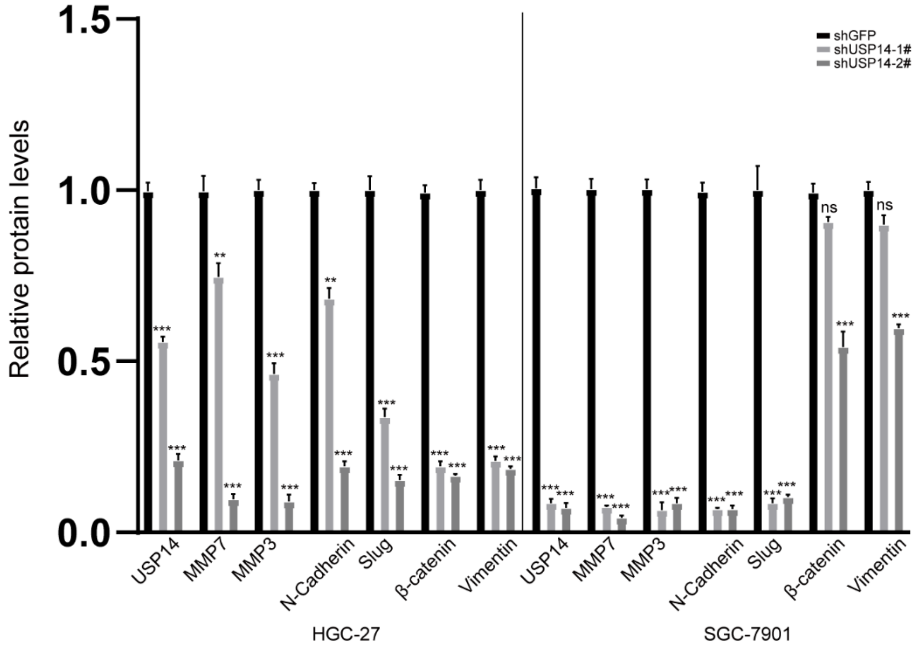

Fig.S4 Western blot assay was used to detect the protein expression levels of metastasis-related proteins in USP14-rescued USP14-knockdown HGC-27 and SGC-7901 gastric cancer cells.

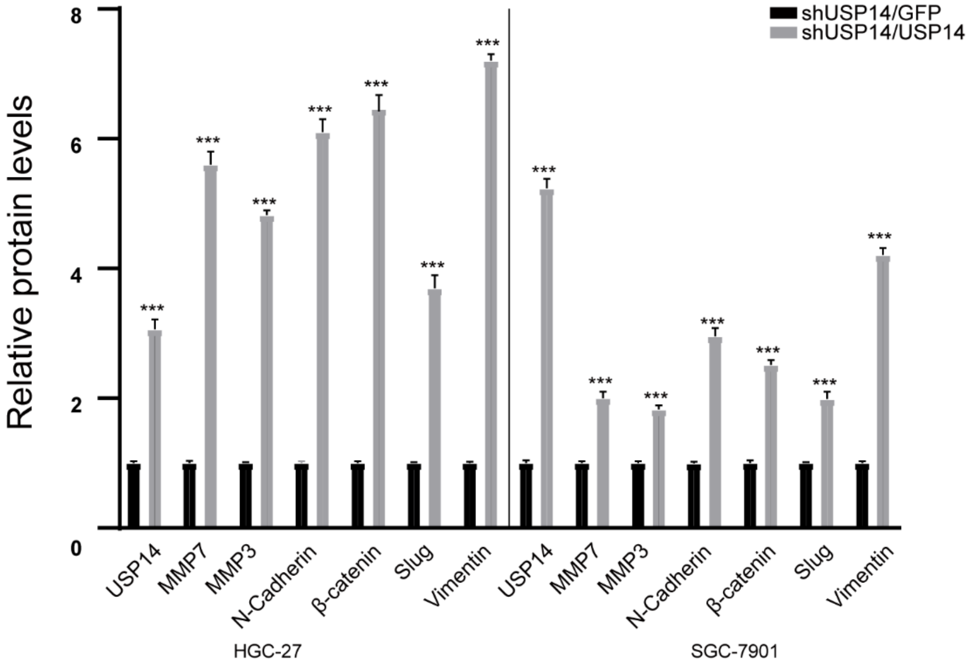

.

Fig.S5 Immunohistochemical staining analysis showed the expression of KPNA2 in different stages of gastric cancer tissues.

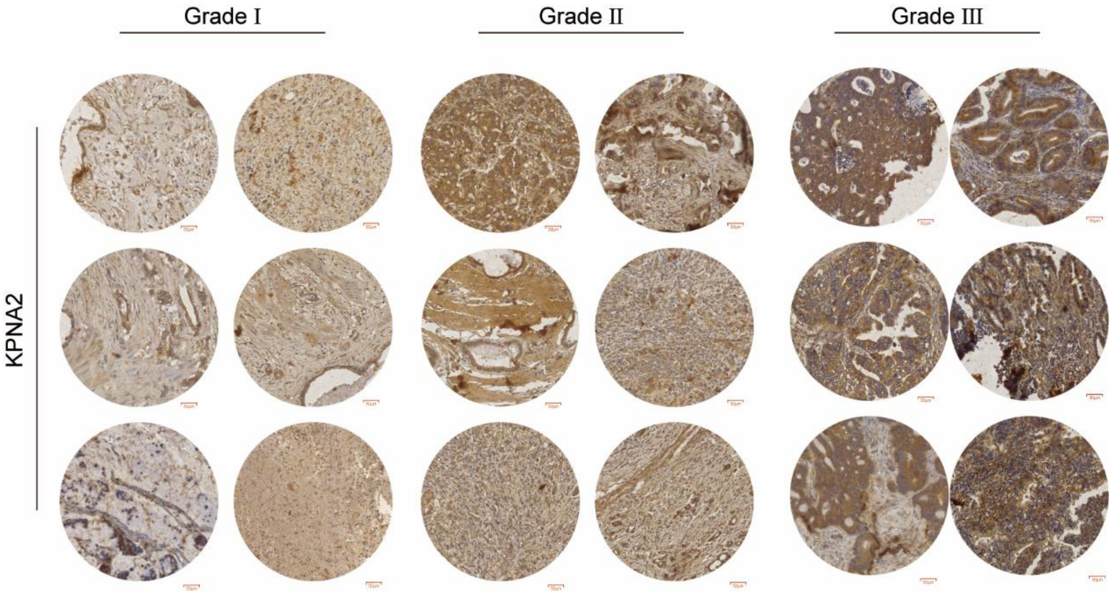

Fig.S6 Knockdown of KPNA2 inhibited proliferation, migration, and invasion of gastric cancer cells.

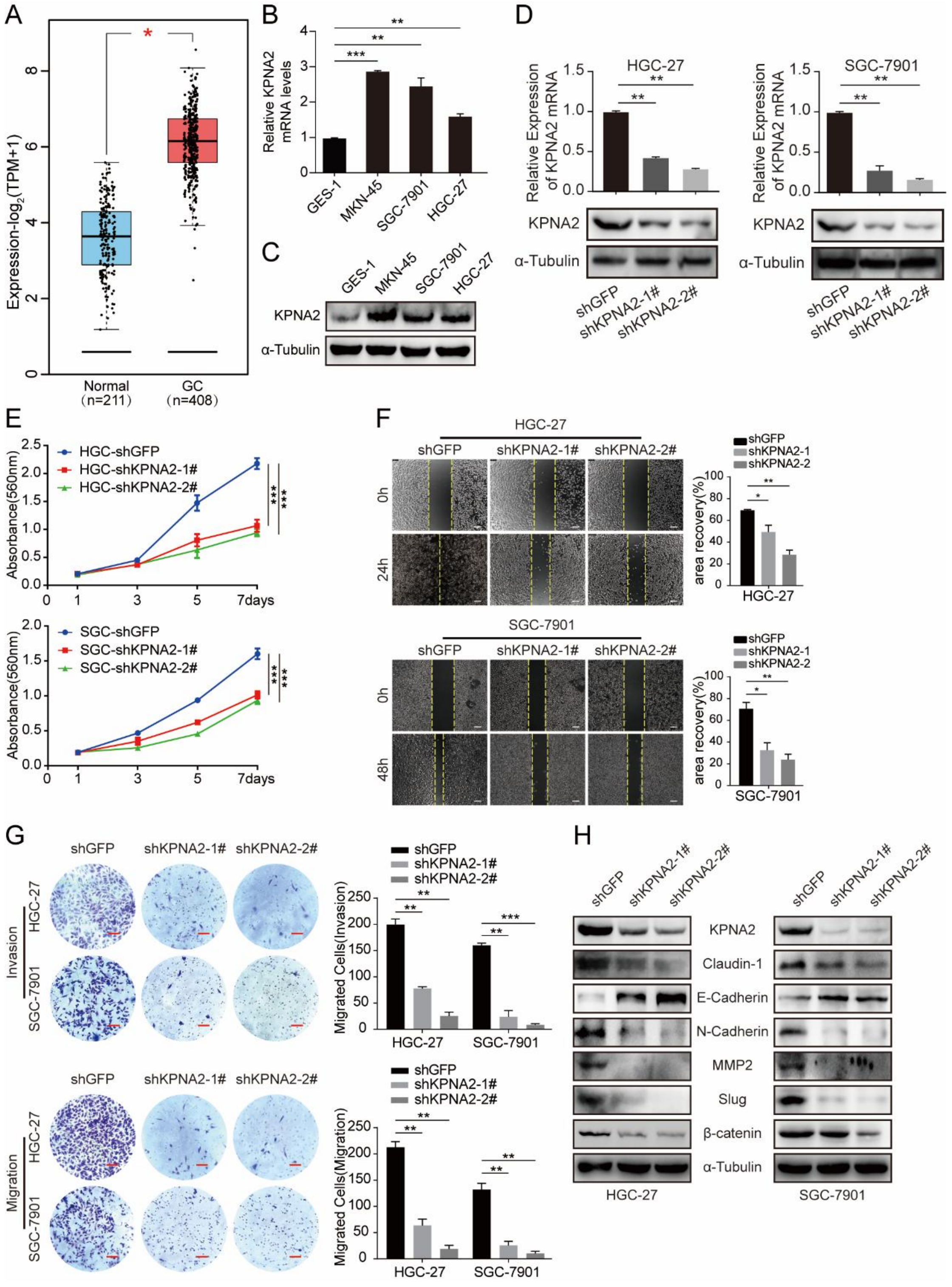

(A) Box plot of KPNA2 expression levels in the peritumoral tissues (normal) and GC tumors with log-rank test P values < 0.05. (B, C) qRT-PCR and Western blot assays were used to detect the protein expression of KPNA2 in the normal gastric cell line (GES-1) and GC cell lines (MKN-45, SGC-7901, HGC-27). (D) Western blot and qRT-PCR assays were performed to characterize the expression of KPNA2 in the control and KPNA2-knockdown HGC-27 and

SGC-7901 cells. (E) MTT assay was performed to test the proliferation of the control and KPNA2-knockdown HGC-27 and SGC-7901 cells. (F, G) Wound healing assay Migration and invasion assays were performed in KPNA2-knockdown cells. Scale bar = 50 μm. Western blot assay was performed to characterize the expression of some key metastasis-related proteins in KPNA2 knockdown cells. All data were expressed as the mean ± SD. Student's t-test was performed to analyze significance. *$P < 0.05$, **$P < 0.01$, ***$P < 0.001$.

Fig.S7 KPNA2 recovery rescues the cell proliferation, migration, and invasion of USP14-silenced GC cells.

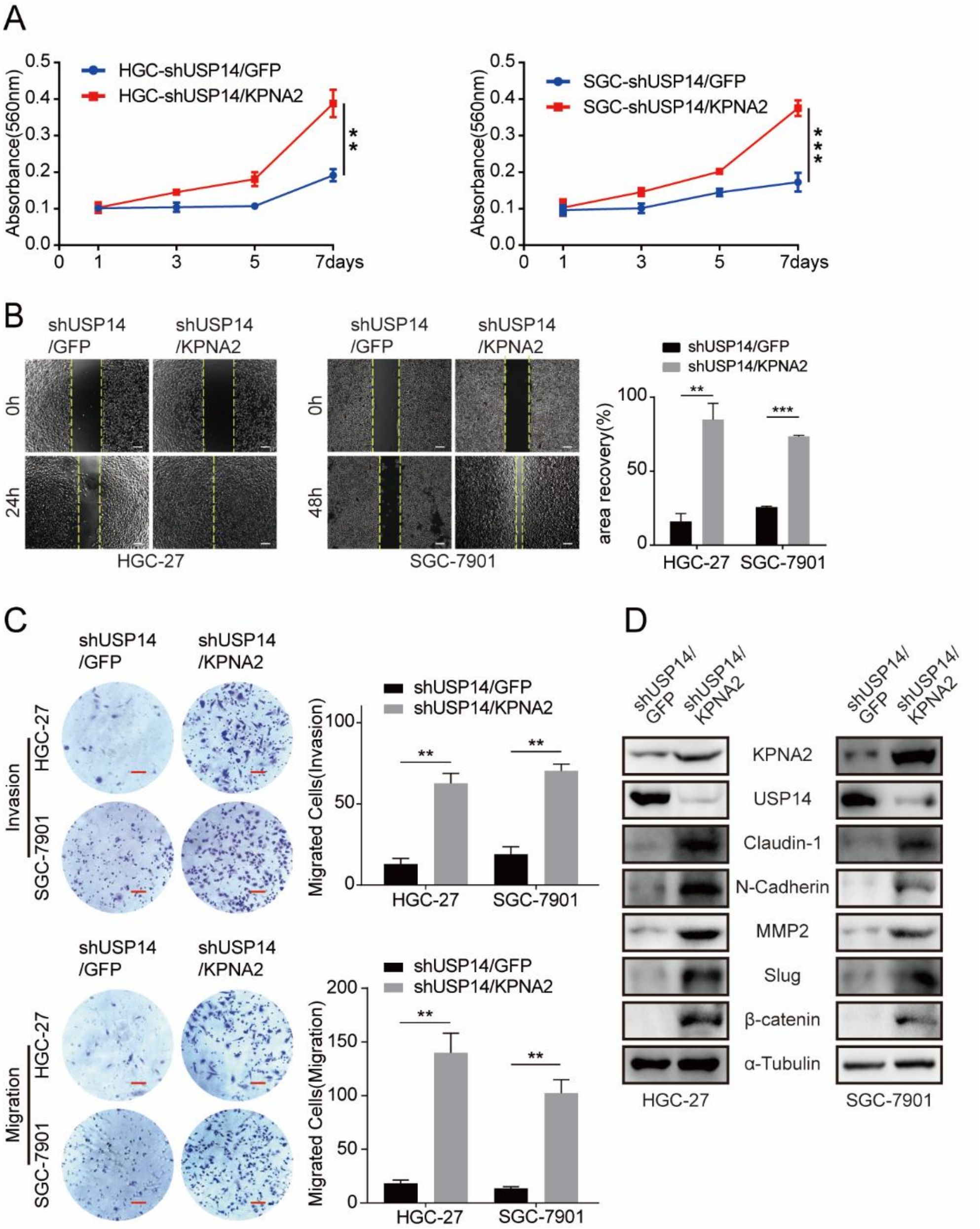

(A) Growth curves were shown for the KPNA2-rescued USP14-knockdown cells. (B, C) Wound healing assay, Migration assay, and invasion assay were performed in KPNA2 rescue USP14-knockdown cells. Scale bar = 50 μm. (D) Western blot assay was performed to characterize the expression of some key metastasis-related proteins in KPNA2 rescue USP14-knockdown cells. All data were expressed as the mean ± SD. Student's t-test was performed to analyze significance. *P < 0.05, **P < 0.01, ***P < 0.001.
