# Supplementary figures and images for: The deubiquitination enzyme USP14 promotes the tumourigenesis of gastric cancer by enhancing c-MYC nuclear translocation through deubiquitination of KPNA2
Source: Cell Death Dis. 2025 Oct 21;16(1):737. doi: 10.1038/s41419-025-08065-2 (PMC12540732; doi:10.1038/s41419-025-08065-2)

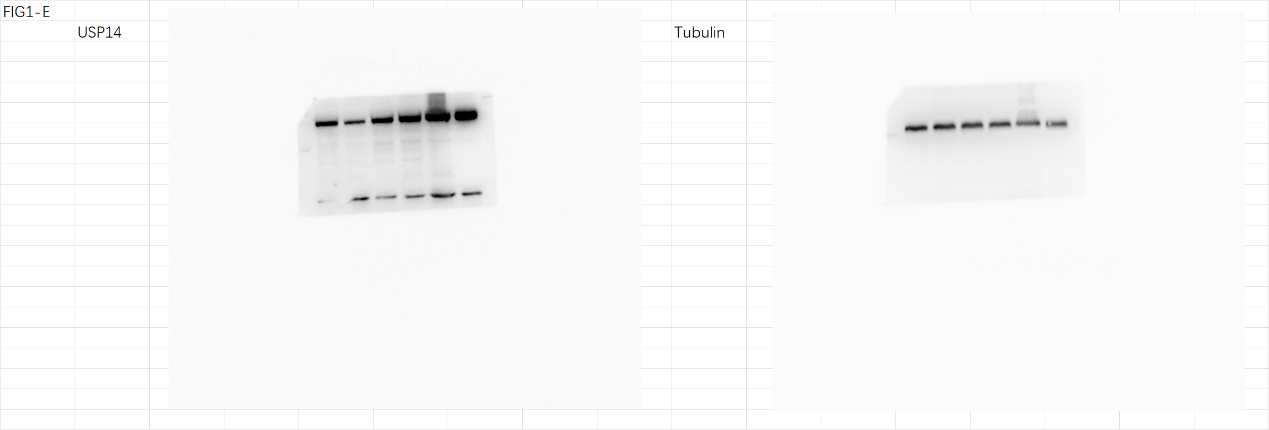


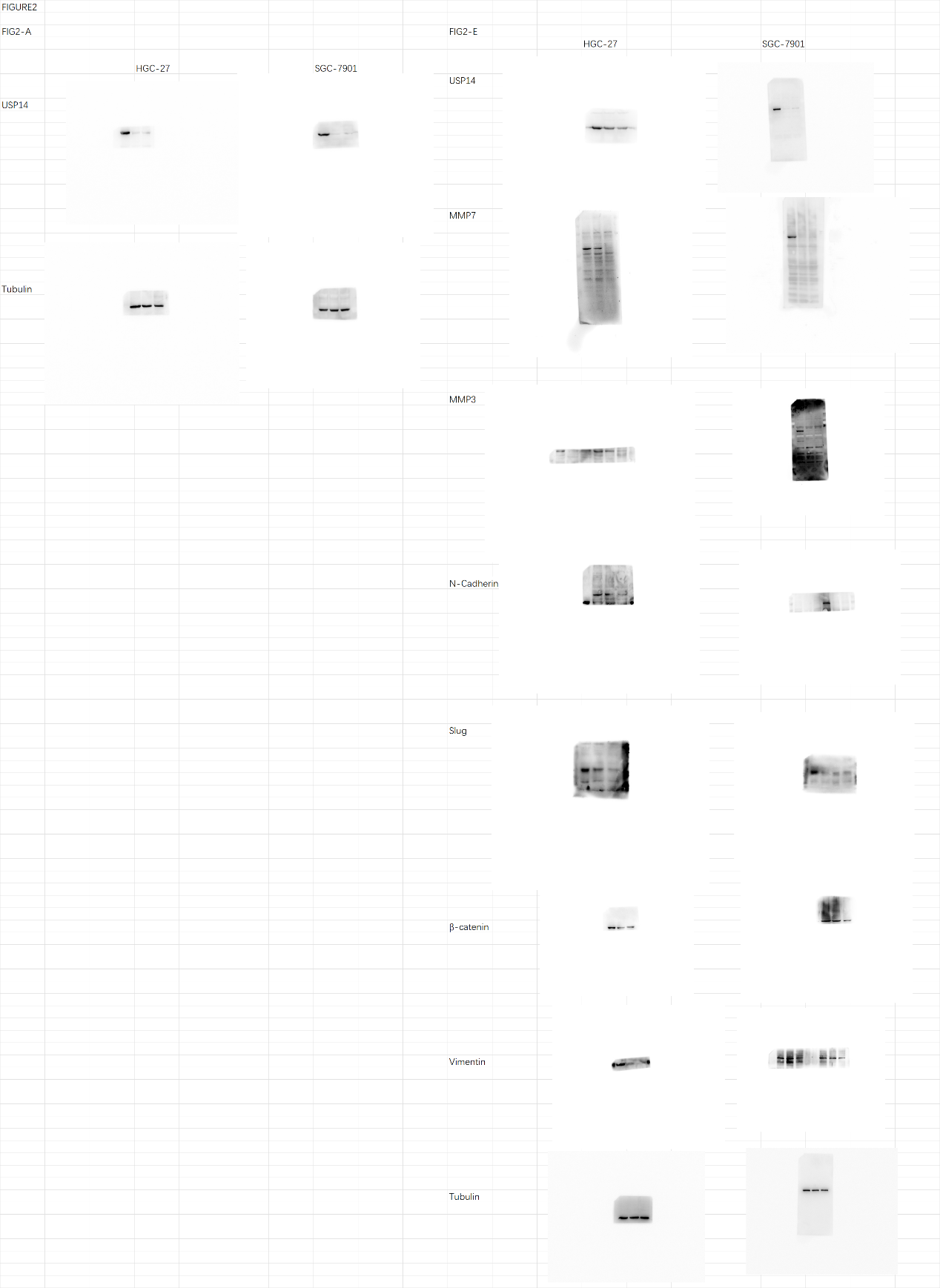


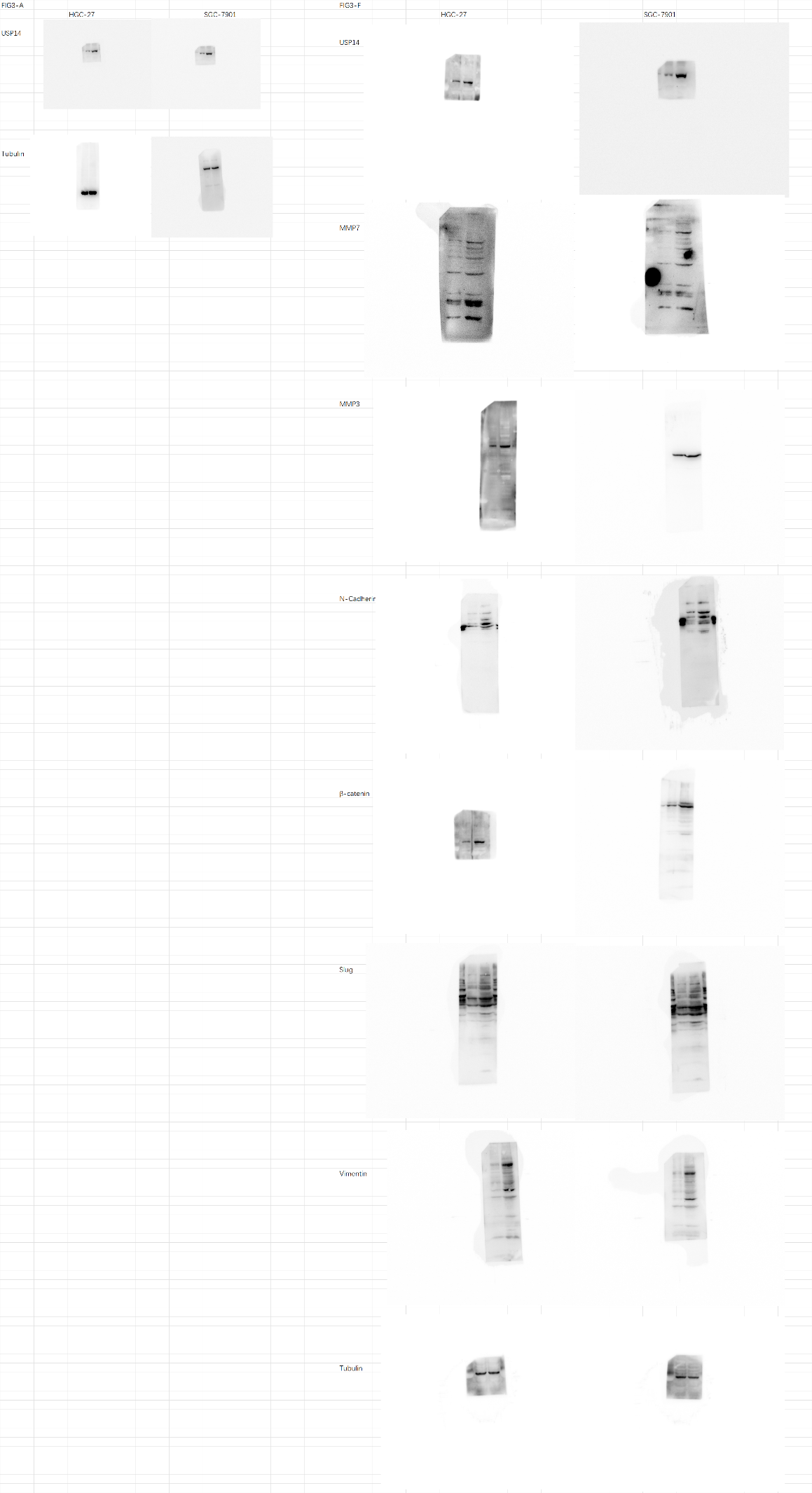

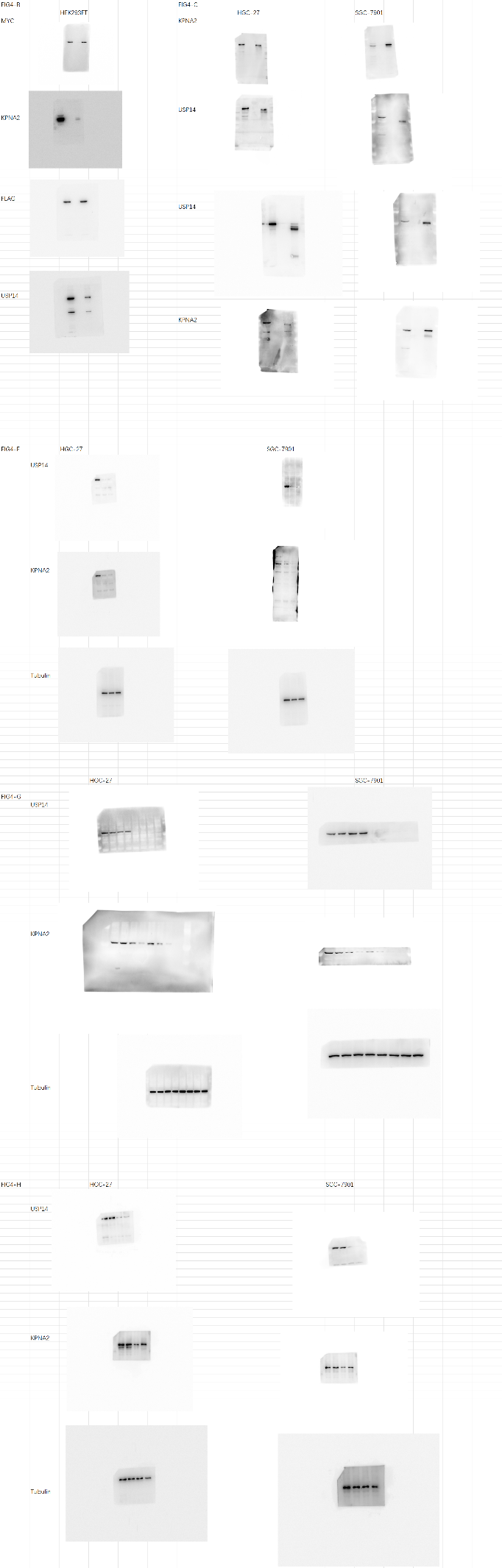

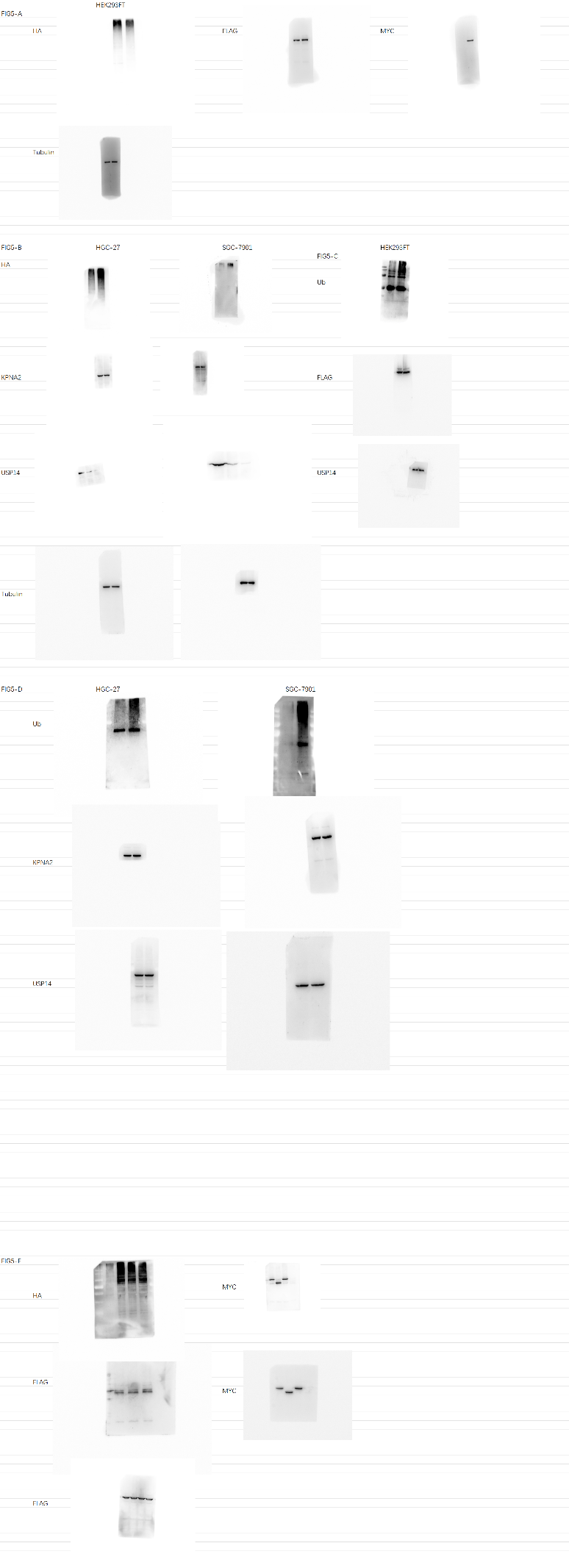

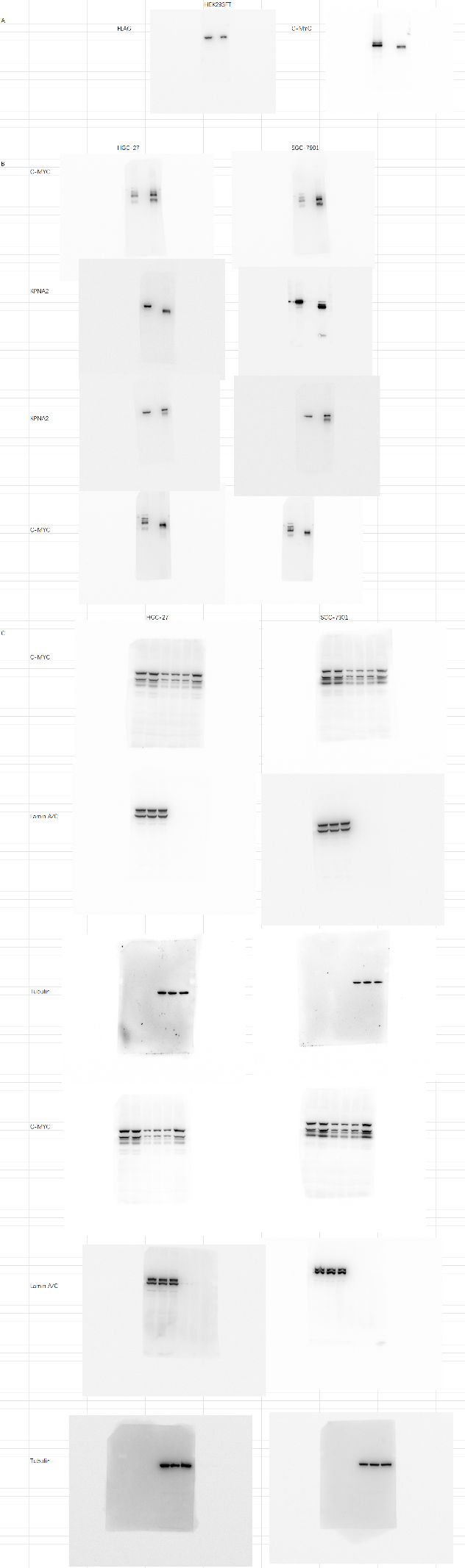

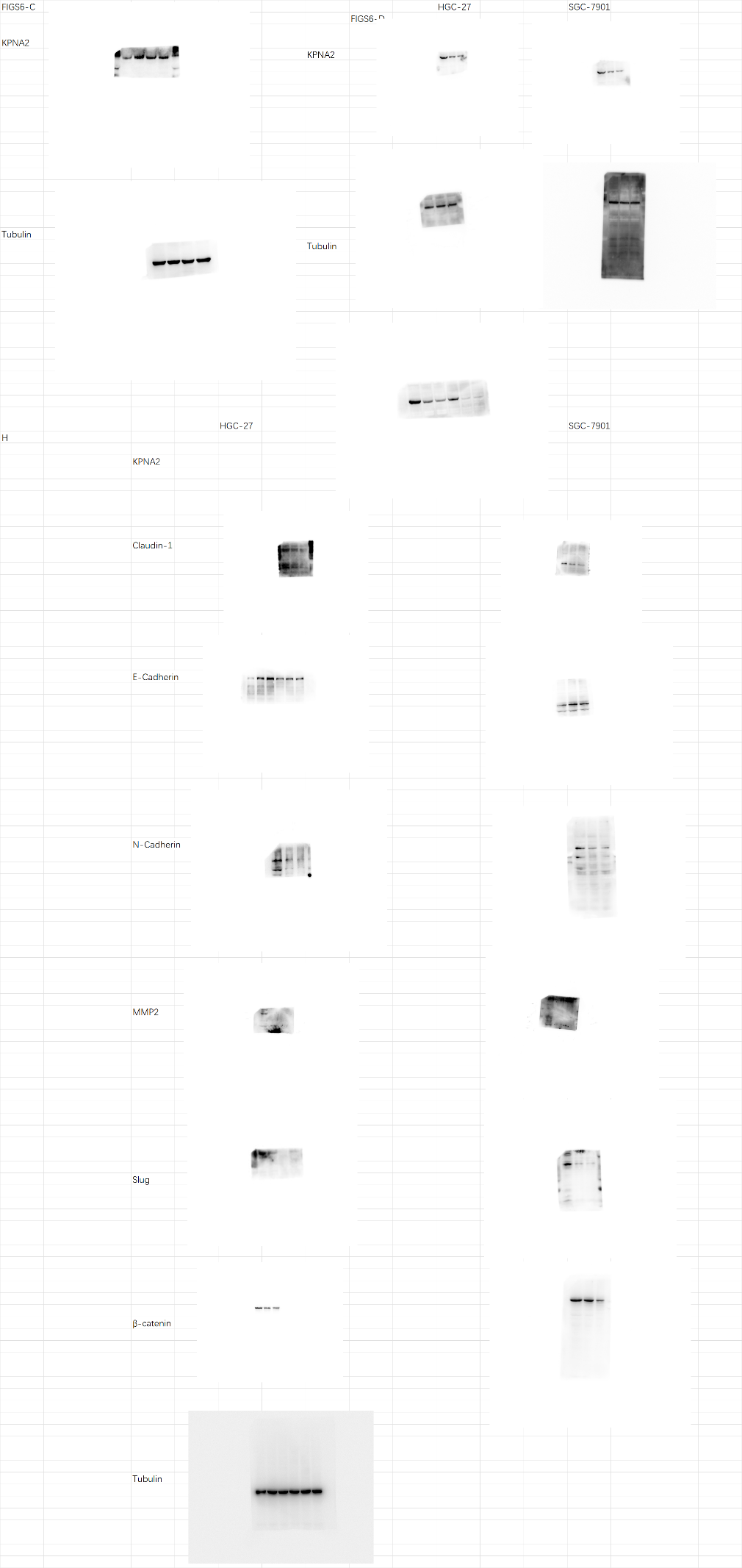

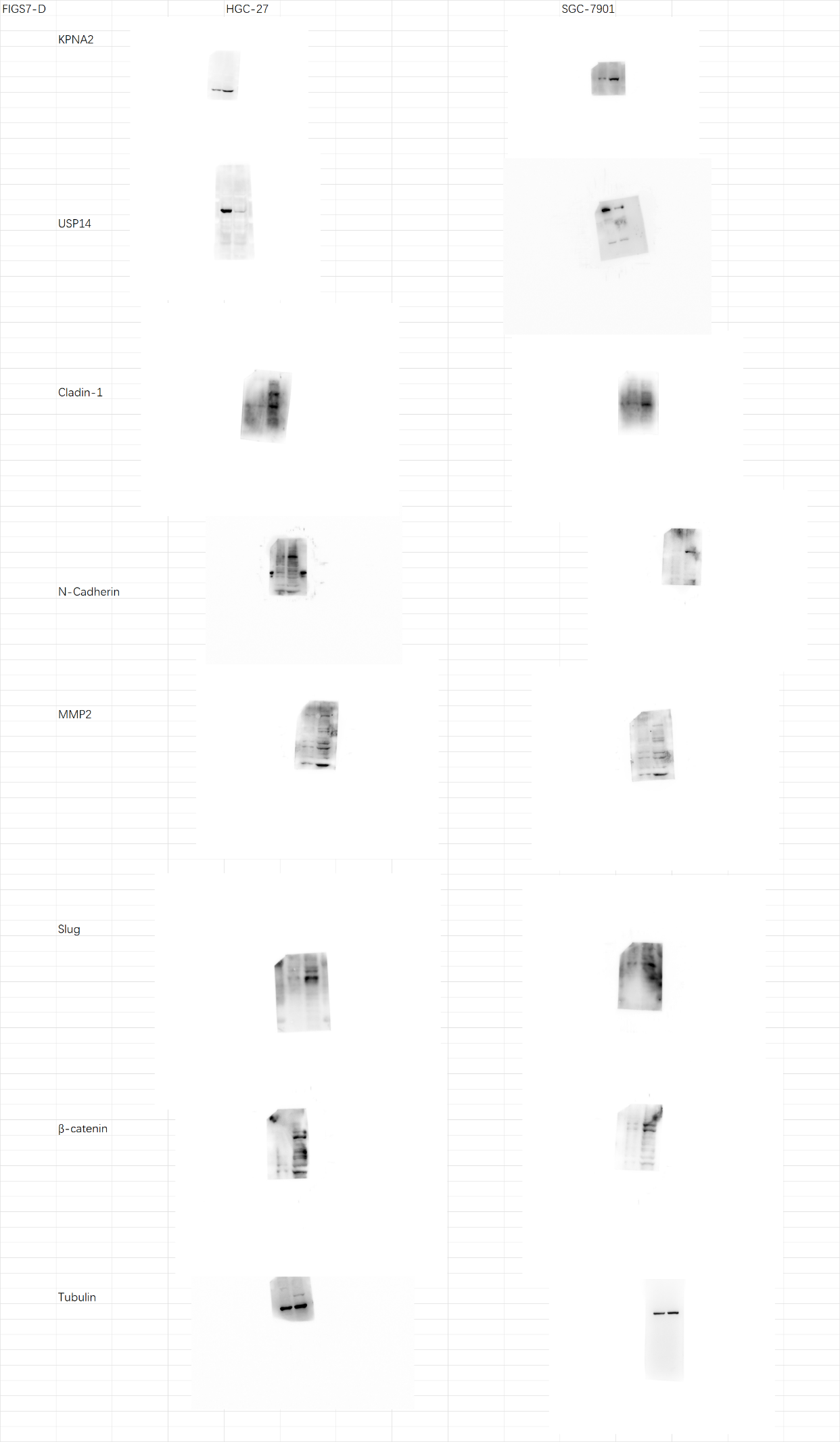

Supplement: Supplementary file 2 — WB RAW DATA [file 41419_2025_8065_MOESM2_ESM.docx]
